# Supplementary material for: Phosphorylation by the stress-activated MAPK Slt2 down-regulates the yeast TOR complex 2
Source: Genes Dev. 2018 Dec 1;32(23-24):1576–90. doi: 10.1101/gad.318709.118 (PMC6295167; doi:10.1101/gad.318709.118)
Supplement: Supplemental Material [file supp_32_23-24_1576__index.html]

Phosphorylation by the stress-activated MAPK Slt2 down-regulates the yeast TOR complex 2 — Supplemental Material 

# Phosphorylation by the stress-activated MAPK Slt2 down-regulates the yeast TOR complex 2

## Supplemental Material

- Supplememental\_Material.pdf
