# Supplementary material for: Phosphorylation by the stress-activated MAPK Slt2 down-regulates the yeast TOR complex 2
Source: Genes Dev. 2018 Dec 1;32(23-24):1576–90. doi: 10.1101/gad.318709.118 (PMC6295167; doi:10.1101/gad.318709.118)
Supplement: Supplemental Material [file supp_gad.318709.118_Supplememental_Material.pdf]

SUPPLEMENTAL FIGURES

Supplemental Figure 1

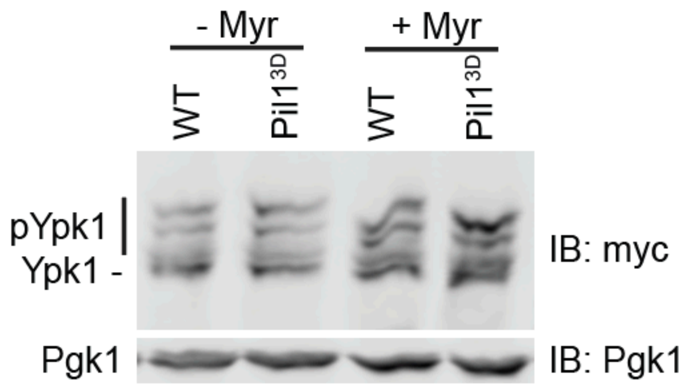

Fig. S1. **TORC2 function is not affected by phospho-mimetic Pil1 allele.** Wild-type (BY4742) or Pil1^{3D} (yEPS2) strains were cultured to mid-exponential phase in selective minimal medium and then treated with vehicle (methanol) or 1.25 µM myriocin for 2 h. After harvesting, whole-cell lysates were prepared, resolved by SDS-PAGE, and analyzed as described in Materials and Methods.

Supplemental Figure 2

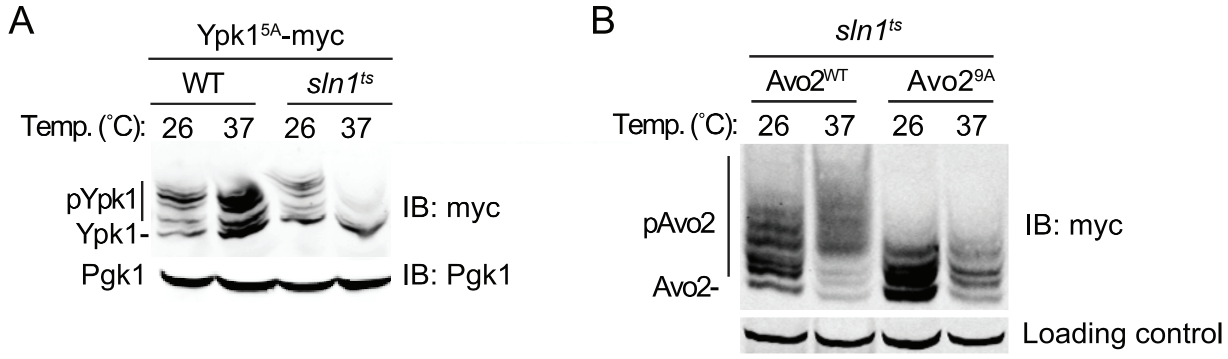

Fig. S2. **TORC2 function is down-regulated after Sln1 inactivation**. (A) Wild-type cells (BY4741) and an otherwise isogenic *sln1^{ts}* derivative (JTY5473) expressing Ypk1^{5A}-myc from its native promoter on a *CEN* plasmid (pFR246) were propagated at 26˚C to mid-exponential phase and then a portion of the culture was maintained at 26˚C and another portion shifted to 37˚C. After 2 h at the respective temperatures, samples of the separate cultures were harvested, lysed and Ypk1 phosphorylation analyzed by Phos-tag SDS-PAGE and immunoblotting. Pgk1 was the loading control. (B) Strain JTY5473 (*sln1^{ts}*) expressing either Avo2-3xFLAG (pKL1) or Avo2^{9A}-3xFLAG (pKL2) were grown to mid-exponential phase and then either kept at 26˚C or shifted to 37˚C. After 2 h, Avo2 phosphorylation was analyzed as in (A).

Supplemental Figure 3

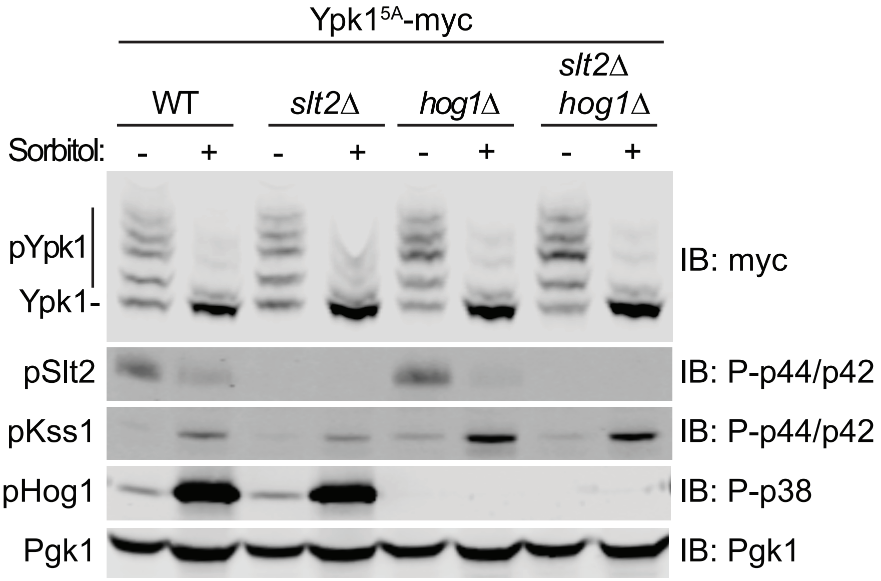

Fig. S3. **Challenge with a high exogenous sorbitol concentration rapidly diminished TORC2-mediated Ypk1 phosphorylation**. Strains JTY5336 (*HOG1^{+} SLT2^{+}*), YFR549 (*slt2*Δ), YFR538A (*hog1*Δ), and YFR559 (*slt2*Δ *hog1*Δ), each expressing Ypk1^{5A}-myc from its native promoter on a *CEN* plasmid (pFR246), were grown to mid-exponential phase, then diluted into fresh SCD-Leu medium in the absence (-) or presence (+) of sorbitol (1 M final concentration). After 5 min, the cells were harvested, lysed and the resulting extracts subjected to Phos-tag SDS-PAGE to resolve Ypk1 phosphorylation and to standard SDS-PAGE to resolve the other proteins, and analyzed by immunoblotting, as described in Materials and Methods.

Supplemental Figure 4

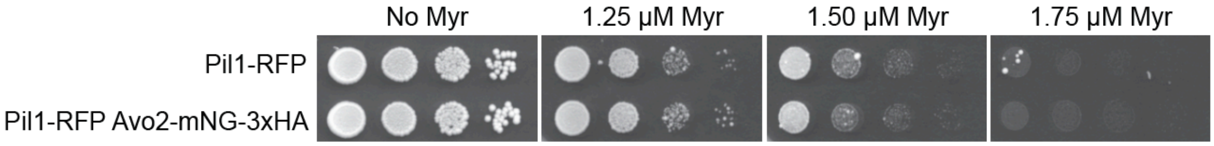

Fig. S4. **Avo2-mNG-3xHA is fully biologically functional.** Overnight cultures of otherwise isogenic Pil1-RFP (yFR181) and Pil1-RFP Avo2-mNG-3xHA (yAEA348) strains were adjusted to A_{600nm} = 0.1, spotted undiluted and in a series of 10-fold serial dilutions on YPD plates either lacking or containing myriocin at the indicated concentrations, incubated at 30°C for 3 d, and then imaged.

Supplemental Figure 5

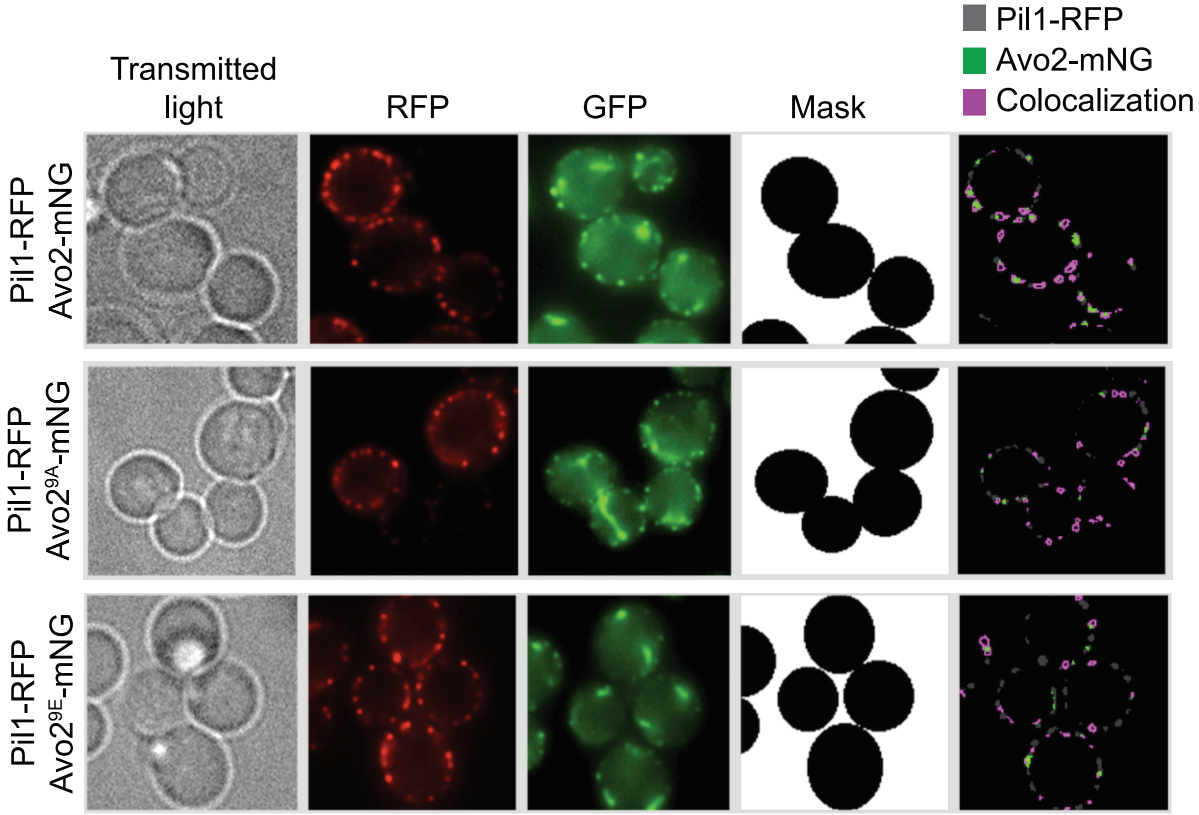

Fig. S5. **Illustration of the masking used to quantify fluorescent images of the cell periphery with CellProfiler**. The images and other data shown in Fig. 5 were obtained using a custom CellProfiler pipeline, as described in Materials and Methods. The blob-like or thread-like cytosolic structures observed in the GFP channel are due to intrinsic autofluorescence of the mitochondria.

Supplemental Figure 6

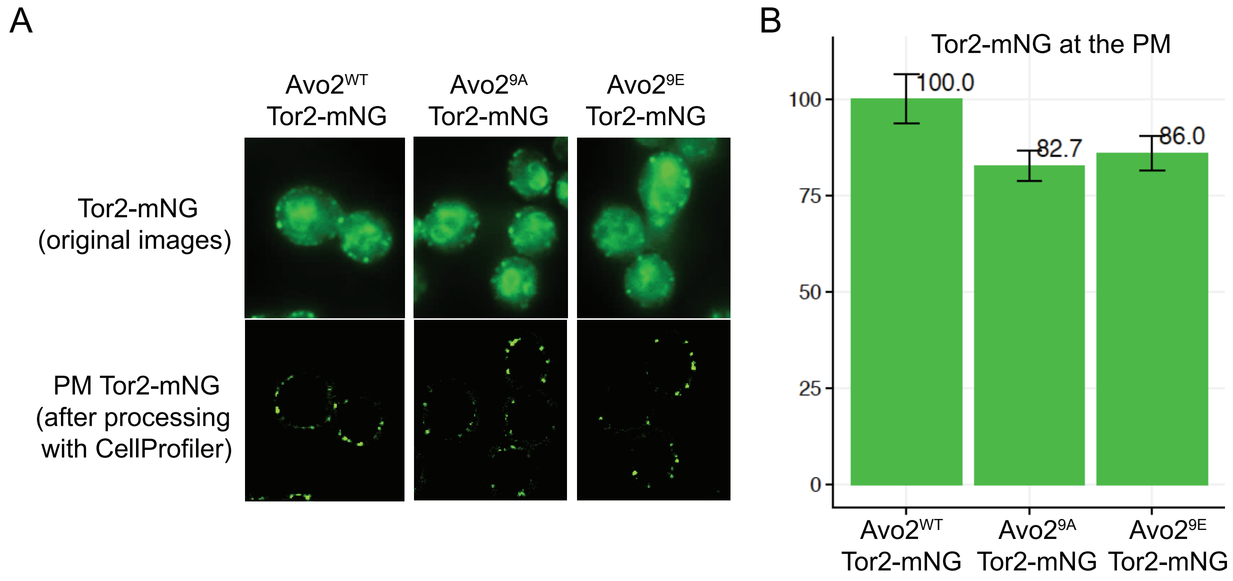

Fig. S6. **The state of Avo2 phosphorylation does not affect Tor2 localization.** (A) Strains YFR624 (Avo2-mKate Tor2-mNG-3xHA Avo3-3xFLAG), YFR626 (Avo2^{9A}-mKate Tor2-mNG-3xHA Avo3-3xFLAG) and YFR628 (Avo2^{9E}-mKate Tor2-mNG-3xHA Avo3-3xFLAG) were grown to mid-exponential phase and examined by fluorescence microscopy as described in Materials and Methods and processed using CellProfiler (as in Fig. S3). (B) Mean values of the relative pixel intensities of the Tor2-mNG foci in images, as in (A), were measured using CellProfiler, and those obtained for strain YFR624 (Avo2-mKate Tor2-mNG-3xHA Avo3-3xFLAG) were set to 100%, to which all the other values were normalized. Error bars represent 95% confidence intervals.
